# New Burmese Amber Rove Beetle Fossils Assigned to the Rare Extant Subfamily Coomaniinae (Coleoptera: Staphylinidae)

**DOI:** 10.3390/insects13090767

**Published:** 2022-08-25

**Authors:** Josh Jenkins Shaw, Alexey Solodovnikov, Ming Bai, Dagmara Żyła

**Affiliations:** 1 Key Laboratory of Zoological Systematics and Evolution, Institute of Zoology, Chinese Academy of Sciences, Beijing 100101, China; 2 Natural History Museum of Denmark, Zoological Museum, Universitetsparken 15, 2100 Copenhagen, Denmark; 3 Leibniz Institute for the Analysis of Biodiversity Change, Zoological Museum, 20146 Hamburg, Germany; 4 Museum and Institute of Zoology, Polish Academy of Sciences, Wilcza 64, 00-679 Warsaw, Poland

**Keywords:** insects‚ beetle, Cretaceous, new species

## Abstract

**Simple Summary:**

The rove beetle subfamily Coomaniinae is incredibly rare, currently comprised of one described and several undescribed species of the genus *Coomania* in Southeast Asia and Northern Australia. Contrary to most rove beetle groups, the subfamily is only known based on a handful of specimens. Coomaniinae is the sister group to the subfamily Staphylininae, which, in contrast, is mega-diverse and abundant in terrestrial habitats globally. In this paper we describe three new species of *Coomania* preserved as fossils from Upper Cretaceous Burmese amber (ca. 99 million years old).

**Abstract:**

The Mesozoic, ca. 99-million-year-old Burmese amber is an incredible source of fossil beetles that have been very actively studied in recent times and have already significantly improved our knowledge about the evolution of the large family of Staphylinidae, the rove beetles. Nevertheless, new extinct taxa of high phylogenetic interest are being discovered, among which the following three rove beetle species are described here: *Coomania megistos* sp. nov., *Coomania enkarsios* sp. nov. and *Coomania yini* sp. nov. These fossils preserved enough morphological characters to be identified as members of the rove beetle lineage formed by Staphylininae and allied subfamilies. Based on the fragments of morphology available for observation, they are hypothesized to be the extinct members of the extant rare monobasic subfamily Coomaniinae, sister to the recently mega-diverse and abundant Staphylininae. Limitations of the available fossil material prompted us to place the new species in the extant, monobasic genus *Coomania* Cameron, 1939, pending a more refined generic placement when more characters become available via additional material or advanced examination techniques. The odd morphology and rarity of the extant members of Coomaniinae restricted to Southeast Asia and Northern Australia make them an enigmatic subfamily among the hyper-diverse Staphylinidae. The newly described fossils, albeit without sufficient details concealed by imperfect preservation, shed some light on the past diversity of Coomaniinae and its divergence from Staphylininae.

## 1. Introduction

Studies in rove beetle palaeontology, especially during the last decade, described over 62 rove beetle species from the Upper Cretaceous Burmese amber [1,2,3,4,5]. New taxa are described at an incredibly high frequency from that deposit [6], which continues to reveal surprises and novel finds, the most recent discovery of the fossil members of the phylogenetically important subfamily Protopselaphinae, which are very rare among the extant fauna, being a good example [7,8].

Thus, it was not unexpected to find fossil inclusions in Burmese amber that resembled the very rare, monobasic and enigmatic recent rove beetle genus *Coomania* Cameron, 1939. *Coomania* is known from a handful of specimens of *C. tonkinensis* Cameron, 1939 from Northwestern Vietnam, Laos and Sabah (Malaysia) [9,10] and one or two more very similar undescribed species from this area and Queensland in Australia. The scarcity of *Coomania* specimens is noteworthy, especially given their very peculiar morphology, which has caused controversies about the systematic placement of this genus.

In his original description, Cameron [9] noted the affinity of his *Coomania* to the genus *Diochus* Erichson, 1839. At that time, both genera were associated with the “Xantholini” and were placed in the tribe Diochini, where they remained until very recently, e.g., [11]. Żyła and Solodovnikov [12], based on the tree topology of their multi-locus molecular phylogeny and taking into consideration the morphological peculiarity of *Coomania*, established a monobasic subfamily, Coomaniinae. In that phylogeny, Coomaniinae was recovered as sister to the subfamily Staphylininae (a clade equivalent to the tribe Staphylinini prior to Żyła and Solodovnikov’s [12] upgrade of classification), in a phylogenetically remote position from the clade of Diochini. Żyła and Solodovnikov [12] argued for a convergent similarity between *Coomania* and Diochini, while establishment of *Coomania* in its own subfamily was supported by an unusual suite of morphological characters that made Coomaniinae distinctive among all rove beetles. Tihelka et al. [13] reanalyzed the data of Żyła and Solodovnikov [12] under a site-heterogeneous CAT-GTR model with distantly related outgroups excluded and also resolved *Coomania* as sister to Staphylinini (Staphylininae sensu Żyła and Solodovnikov [12]), phylogenetically away from Diochini. Within their more inclusive concept of Staphylininae, Tihelka et al. [13] treated *Coomania* as its own tribe, Coomaniini, sister to the tribe Staphylinini. The large morphological dissimilarity between the recently rare *Coomania* (Coomaniinae) and its sister clade of recently mega-diverse Staphylininae (=Staphylinini sensu Tihelka et al. [13]), as well as the geological age of Staphylininae documented by Early Cretaceous fossils [14] suggest a long and heterogenous evolution of these two sister clades, in particular the presumed extinction of stem lineages with the intermediate morphology between them.

In this respect, the three newly found fossil species from 99 Ma Upper Cretaceous Burmese amber that match the diagnosis of recent Coomaniinae, as far as their partially preserved characters permit, are noteworthy for taxonomic study. Here we describe them and discuss these new findings. To place the fossil species into the context of the group hitherto known only from the outdated original description of the genus and subsequent molecular-based phylogenetic work, we provide necessary morphological details about recent species of *Coomania* too.

## 2. Materials and Methods

This study is based on four pieces of Burmese amber deposited at the Shanghai Normal University (Shanghai, China). The pieces of amber are from Hukawng Valley, Kachin State, northern Myanmar. UePb zircon dating suggests that the age of the amber is Late Cretaceous (earliest Cenomanian, ca. 99 Ma) [15]. Measurements were made using ImageJ [16]. The following measurements were taken: HL (Head length); EyL (Eye length at longest point); PW (Pronotal width at widest point); EL (Elytral length from apex of mesoscutellum to end of elytral suture); EW (Elytral width at widest point). For some species certain measurements were omitted due to difficulty obtaining them due to fossil preservation.

Amber pieces were studied using Olympus SZ61 and Leica M205C stereomicroscopes. Comparative extant material came from the Natural History Museum of Denmark (NHMD), Field Museum of Natural History (FMNH), Natural History Museum of Geneva (MHNG), Natural History Museum Vienna (NHW). Photographs were taken using a Canon EOS 5D with Canon MP-E 65mm macro lens, a Canon EOS 6D and a Leica M205 C stereomicroscope and a Canon EOS 6D DSLR digital camera mounted on a Zeiss Axioskop 50 via an LM Digital SLR Universal Adapter. Photographs were stacked using ZereneStacker 1.04 (Zerene Systems LLC, Richland, USA). Photographs were edited using Adobe Photoshop CS6 (Adobe Systems, San Jose, CA, USA).

## 3. Results

### 3.1. Taxonomy of Recent Coomaniinae

#### 3.1.1. Composition

**Figure 1:**
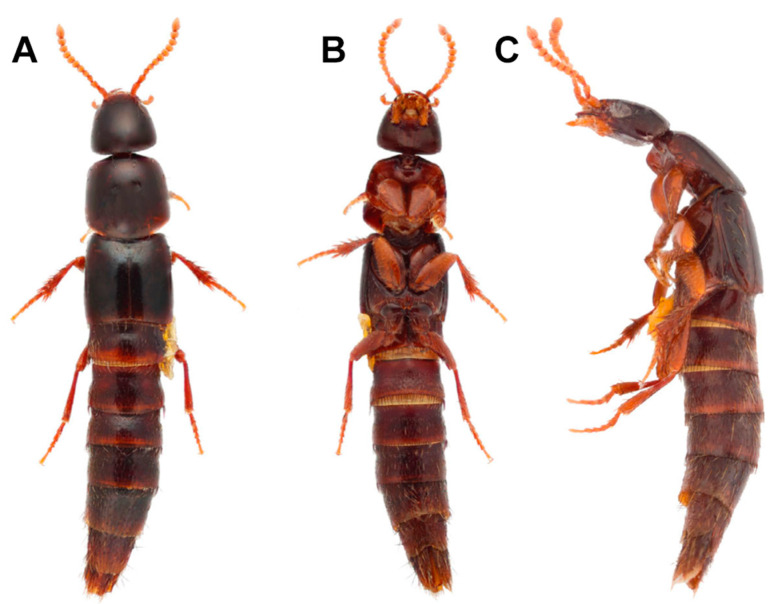
*Coomania* sp. Habitus: (**A**), dorsal view. (**B**), ventral view. (**C**), lateral view. Reproduced from Żyła and Solodovnikov [12].

The genus contains *C*. *tonkinensis* Cameron, 1939, described in northwestern Vietnam (Cameron, 1939) and later reported from Laos and Sabah (Rougemont, 2018). We are aware of several more specimens from Laos, Malaysia, Philippines and Queensland in Australia, externally nearly identical to *C*. *tonkinensis* but presumably belonging to one or two undescribed species pending a taxonomic revision (one undescribed specimen photographed in figure 5A–C in Żyła and Solodovnikov [12], reproduced in Figure 1A–C).

#### 3.1.2. Morphology of the Extant Members of the Genus Coomania

*Coomania tonkinensis* and its undescribed recent relatives are medium-sized rove beetles of ca. 6 mm long with a rather cylindrical dark brown glossy body without distinct microsculpture or punctation (Figure 1A). Head about as wide as long; temples (in dorsal view) diverging towards abruptly truncate basal margin of head (Figure 1A); eyes small, nearly two times as short as temples; gular sutures widely separated from each other along their entire length; neck very narrow, at most 1/4 of head width; no clear ridges on head or around neck region; labrum about as wide as long, apically incised, with apical setae, without apical membrane; mandibles short, sharp, symmetrical, gradually curved with strong inner tooth each; apical maxillary palpomere aciculate, penultimate wide and glabrous; apical labial palpomere nearly aciculate, ca. two times as thin as penultimate palpomere; antennal bases concealed under the “shelf” of frons, thus not visible from above; antennae attached closer to eye than to each other, robust, with first segment about as long as second and third segments combined, the following antennomeres gradually becoming shorter and wider, subapical antennomeres slightly transversal, apical antennomere ca. two times longer than wide. Prothorax with fully sclerotized ventral side, also behind coxae with spiracles embedded into this sclerite; inferior marginal line not traceable, superior marginal line not bending under anterior margins of pronotum; pronotal hypomera inflexed but slightly visible posteriorly in lateral view; prosternum with slight longitudinal carina between anterior coxae, without antesternal plates; a disc of pronotum with pair of punctures slightly before the middle. Elytra without distinct hypomera, with sub-basal ridge very distinct, extending from mesoscutellum to humerus; mesoctutellum with one basal carina; mesosternum anterior to middle coxae with a weak sub-basal ridge. Legs robust, tarsal formula 5-5-5, all tarsi with pair of short empodial setae; anterior tarsi broadened (more in males than in females) with adhesive setae ventrally; all tibia with strong spurs and spines; posterior coxae clearly divided into a bulgy smooth basal part and more flat apical part with setation. Abdomen cylindrical, with clear protergal glands (figure 5D in Żyła and Solodovnikov [12], reproduced in Figure 1A–C); all segments with one basal carina; segment III with pair of narrow paratergites, other segments without paratergites, sternites fully fused with tergites forming a ring; sternite III with sharp longitudinal carina between coxae; lateral tergal sclerites IX large in both sexes, especially in males, apically not inflated, fully separated from each other at base by tergite X (figure 5E in Żyła and Solodovnikov [12]); female sternite IX consists of one pair of well sclerotized gonocoxites (presumably apical gonocoxites) fully separated from each other by weaker sclerotized triangular central sclerite (presumably fused basal gonocoxites); male sternite IX absent (figure 5F in Żyła and Solodovnikov [12]); sternite VIII clearly apically incised in males; intersegmental membrane with brick-wall-like sclerites; aedeagus highly modified, with extremely enlarged parameres embracing highly reduced and desclerotized median lobe [or parameres absent, if sclerotized body of aedeagus interpreted as median lobe] (figure 5G,H in Żyła and Solodovnikov [12]). Larvae are unknown.

### 3.2. Systematic Palaeontology

Order Coleoptera Linnaeus, 1758;

Family Staphylinidae Latreille, 1802;

Subfamily Coomaniinae Żyła and Solodovnikov, 2020;

Genus *Coomania* Cameron, 1939.

#### 3.2.1. Placement of the Fossils

The new fossil species described here can be placed in the extant subfamily Coomaniinae because of the certain habitus similarity, lacking other sound alternatives and the following shared diagnostic combination of characters unique to Coomaniinae: antennal bases concealed under “shelf”; neck very narrow; mesosternite with sub-basal ridge present; abdominal segments IV to VIII without paratergites; mesotibiae with multiple spurs. Unfortunately, imperfect preservation of the fossils conceals the ventral side of the thorax, which in recent Coomaniinae is very special due to full sclerotization of the area behind the coxae and a lack of antesternal plates anteriorly. Moreover, the terminalia and aedeagus, which are highly peculiar in this subfamily, are unavailable for study in our fossils. Among other rove beetles, the absence of the paratergites is characteristic of Osoriinae and may be found in some Euaesthetinae, Steninae and the subtribe Procirrina of the subfamily Paederinae (Pinophilinini). From all these, our fossils can be easily distinguished as follows: from Osoriinae (which similarly to our fossils have antennal bases concealed under frons “shelf”) at least by a very narrow neck (usually very wide in Osoriinae) and by more narrow mandibles without robust molar area (trait well visible in *Coomania encarsios* here), as well as by tarsal formula 5-5-5 with enlarged anterior tarsi (often 4-4-4 or 3-3-3 with all tarsi narrow in Osoriinae); from Steninae at least and easily by very different habitus without big eyes, etc.; from Euaesthetinae at least and easily by not clubbed antennae and not slender, not falcate mandibles; from Procirrina at least and easily by the lack of expanded maxillary palpomere 4 and expanded protarsomeres that define that subtribe of Paederinae.

Within the sister group Staphylininae, the fossils significantly resemble members of the tribe Diochini. This similarity is enforced by the structure of the mandibles, which are visible in *Coomania enkarsios* and which are straighter and without strong internal teeth, as in recent *Diochus* (curved and with a strong internal tooth in recent *Coomania*). However, from species in the tribe Diochini, our fossils differ by the absence of paratergites (always distinct in Diochini). Additional characters that could be informative for placement of these fossils, especially in comparison with Diochini (e.g., antesternal area with or without plates, postcoxal area of the hypomeron with or without process, sclerotized or not), unfortunately, are not visible in them due to imperfect preservation. Significant morphological diversity is observed among three fossil species described here and some, even in habitus, a difference from the extant *Coomania* suggests that they may be non-congeneric. With the limited material currently available, their placement in the recent genus *Coomania* seems a more appropriate solution.

#### 3.2.2. Descriptions of New Species

**Figure 2:**
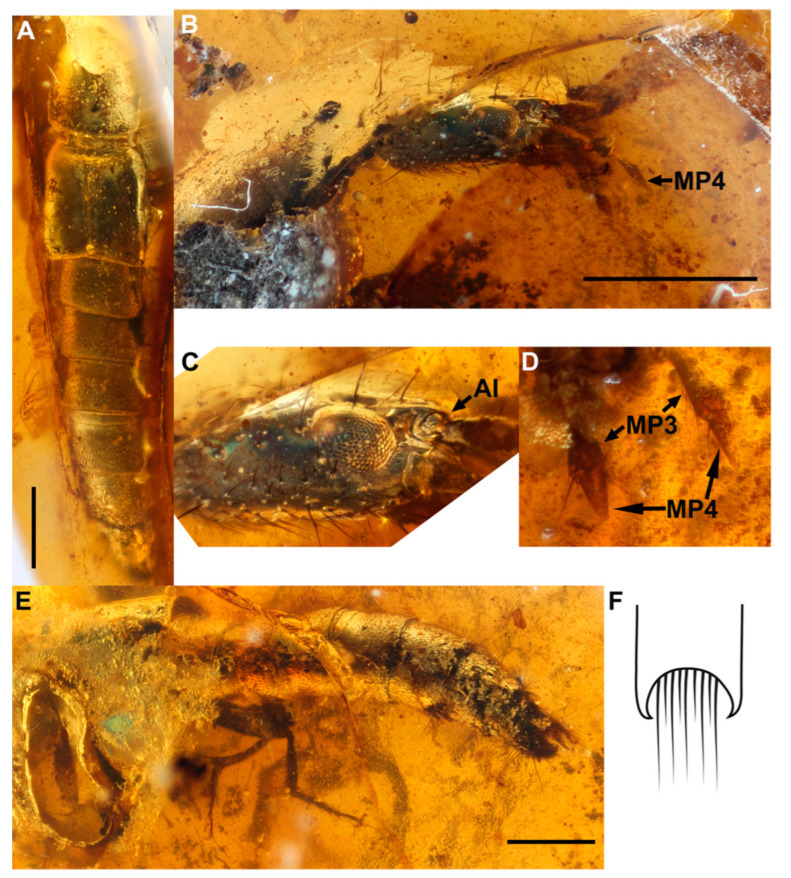
*Coomania megistos* sp. nov. Holotype SNUC-Paleo-0104. (**A**), dorsal view. (**B**), right side of head in lateral view. (**C**), close up of right side of head. (**D**), maxillary palpi. (**E**), left side of abdomen in lateral view. (**F**), drawing of tergite IX in dorsal view. Scale bars-1 mm. AI-antennal insertions; MP-maxillary palpomere.

*Coomania megistos***sp. nov.** (Figure 2)

urn:lsid: urn:lsid:zoobank.org:act:27D93A64-0724-435A-8BB6-643CC551F405

Type material: Holotype SNUC-Paleo-0104.

Diagnosis: *Coomania megistos* sp. nov. differs from all extant and extinct congeners in its large size and the conical shape of the apical maxillary palpomeres.

Description: large, robust beetle, rather parallel-sided along its length, black body. Approximately 9 mm in length (Figure 2A): HL: 1.4; EyL: 0.28; PW: 1.1; EL: 0.96; EW: 1.3 Head: eyes occupying just under third of side of head; temples approximately twice length of eyes (Figure 2B,C); neck narrow. Antennae: antennal insertions under “shelf” and therefore presumably not visible in dorsal view (Figure 2B,C; AI). Maxillary palpomere 4 conical, slightly shorter and thinner than maxillary palpomere 3 (Figure 2D). Ventro-lateral side of head with line of closely positioned setae, all directed anteriad towards mouthparts (Figure 2B,C). Prothorax: basisternum with longitudinal sharp keel; pronotum appearing metallic; with shallow furrow along mid-line; pronotum with hind angles rounded in dorsal view (Figure 2A). Protarsomeres 1 to 4 strongly expanded with dense setae ventrally; protarsomere 5 elongated without setae ventrally. Mesothorax: Mesoscutellum impunctate and glabrous with weak transverse microsculpture. Elytra appearing metallic; elongate (Figure 2A), forming obtuse angle at posterior tip of elytra suture; glabrous and unpunctured, laterally with row of ca. 12 setae; area immediately on either side of elytral suture weakly raised, creating weak furrow containing ca. 6 macrosetae along length of elytral suture; sub-basal ridge (sbr) present, extending from mesoscutellum to humerus. Metafemora rather densely setose with transverse microsculpture. Metatibiae with two spurs in apical third of inner surface and single spur apically. Abdomen cylindrical (Figure 2E); matt due to fine transverse microsculpture; segment III with single paratergite connecting tergite III to sternite III on each side; remaining segments without paratergites or suture dividing tergites and sternites; abdominal segments covered in adpressed setae as well as several longer, erect macrosetae; intersegmental membrane with fine brick-wall pattern; tergite IX deeply emarginate (Figure 2E), apical tips acutely hooked and directed upwards and slightly inwards.

Etymology: The species epithet is derived from the Greek word “μέγιστος (mégistos)” meaning “biggest” in reference to this species being the biggest species among extinct and extant *Coomania*.

Remarks: The right side of the beetle, especially the pro-, meso-, metathorax and abdomen are obscured by organic matter.

**Figure 3:**
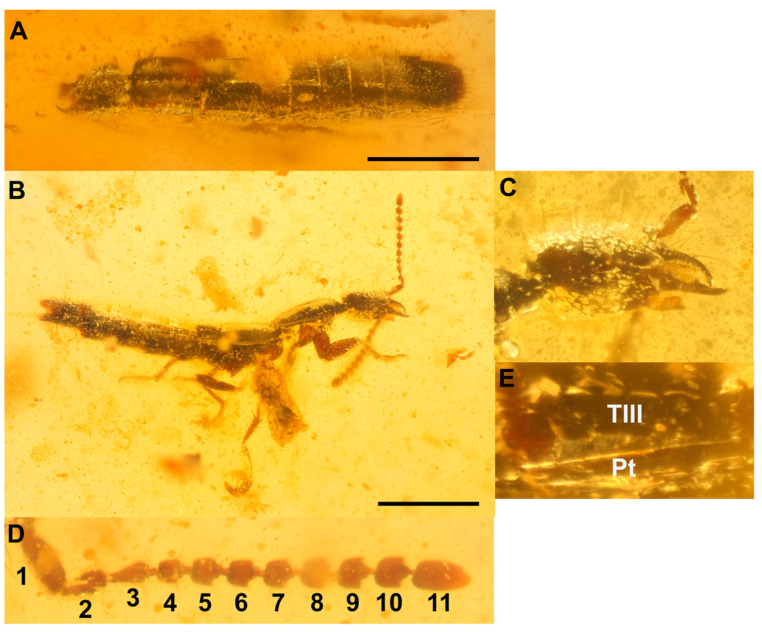
*Coomania enkarsios* sp. nov. Holotype SNUC-Paleo-0105. (**A**), dorsal view. (**B**), lateral view. (**C**), close up of head and mandibles. (**D**), right antenna. (**E**), tergite III with paratergite. Scale bars-1 mm. TIII-tergite III; Pt-paratergite.

**Figure 4:**
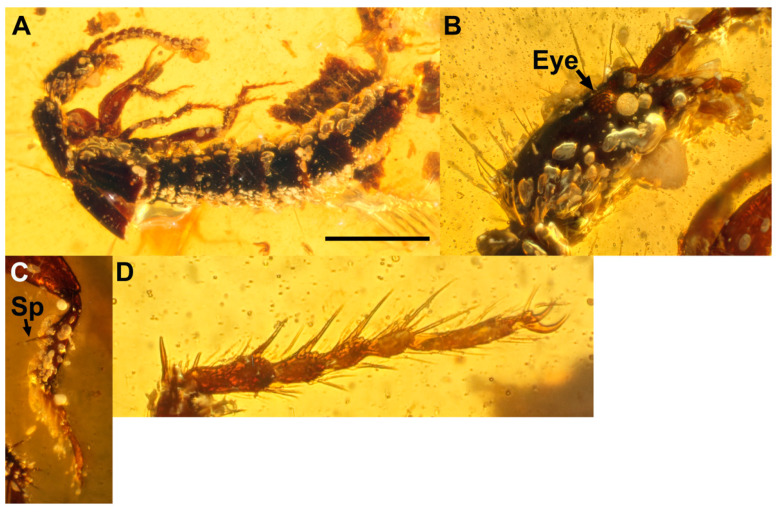
*Coomania enkarsios* sp. nov. Paratype SNUC-Paleo-0106. (**A**), lateral view. (**B**), close-up of head. (**C**), front leg. (**D**), right metatarsus. Scale bar-1 mm. Sp-spur.

*Coomania enkarsios***sp. nov.** (Figure 3 and Figure 4).

urn:lsid: urn:lsid:zoobank.org:act:CA4B685F-3C52-4C99-9028-9603D3E376C7.

Type material: Holotype SNUC-Paleo-0105. Paratype SNUC-Paleo-0106.

Diagnosis: *Coomania enkarsios* sp. nov. differs from all extant and extinct congeners in its small size; antennomeres 5 to 10 transverse; distinctly produced mandibles; the shape of antennomere 1 (fusiform; widest in the middle).

Description: Small, rather delicate beetle (Figure 2A,B and Figure 4A), with black body and uniformly brown palpi, antennae and legs. Approximately 3.5 mm in length (Figure 3A,B and Figure 4A). HL: 0.4; PW: 0.5; EL: 0.4. Head: posterior margin of head in lateral view characteristically “sheared off” with posterior side and ventral side at ca. 120°; Head: eyes occupying 1/4 of side of head (Figure 4B); neck narrow. Mandibles produced; right mandible is more strongly curved than left mandible and slightly de-flexed ventrally (Figure 3A–C). Antennae (Figure 3D) uniformly brown: antennomeres 1 to 3 elongate; antennomere 1 as long as antennomeres 2 and 3 combined, fusiform in shape, widest in middle (Figure 3C) antennomere 4 roughly quadrate; antennomeres 5 to 10 gradually becomes strongly transverse; antennomere 11 elongate. Prothorax; pronotum sinuate in lateral view, superior marginal line apparently inflexed under front angles and therefore not visible in dorsal view. Protibiae with large spur on inner margin, approximately halfway along length of tibia (Figure 4C; Sp); slightly shorter spur present on apical area of protibia. Protarsomeres 1 to 4 expanded with dense setation ventrally (Figure 4C); protarsomere 5 elongate, about as long as protarsomeres 2 to 4 combined with several sparsely positioned setae ventrally. Mesothorax: mesoscutellum impunctate and glabrous. Elytra glabrous and unpunctured, laterally with row of ca. 5 setae; sub-basal ridge (sbr) present. Mesotibiae with large apical spurs and additional spurs along length of mesotibiae. Metatarsi (Figure 4D); metatarsomere 1 as long as metatarsomeres 2 and 3 combined; metatarsomere 5 almost as long as metatarsomeres 2 to 4 combined. Abdomen cylindrical (Figure 4A), especially so from abdominal segmented V; segment III with single paratergite connecting tergite III to sternite III on each side (Figure 3E); paratergite wedge-shaped, widest anteriorly and gradually becoming narrow towards posterior edge of tergite III; remaining segments without paratergites or suture dividing tergites and sternites; apical area of tergites III to VIII dark red (particularly visible on paratype SNUC-Paleo-0106). Apical segments of abdomen covered with long erect setae.

Etymology: The species epithet is derived from the Greek word “εγκάρσιος (enkársios)”, meaning “transverse”, in reference to this species’ having antennomeres 5 to 10 transverse to strongly transverse.

**Figure 5:**
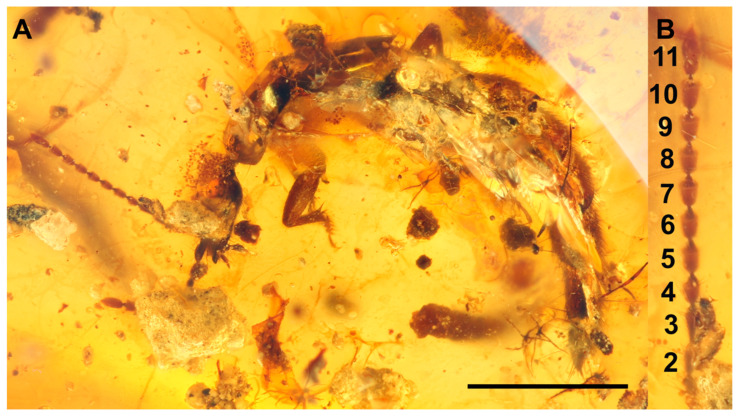
*Coomania yini* sp. nov. Holotype SNUC-Paleo-0107. (**A**), lateral view. (**B**), antennae. Scale bars = 1 mm.

*Coomania yini***sp. nov.** (Figure 5).

urn:lsid: urn:lsid:zoobank.org:act:1710CA29-180D-4AC5-AA3B-18363C5A67BF.

Type material: Holotype SNUC-Paleo-0107.

Diagnosis: *Coomania yini* sp. nov. differs from all extant and extinct congeners in a combination of its rather small size and all antennomeres elongate; antennomere 1 expanded, widest apically.

Description: Rather small beetle, with black body and uniformly brown palpi, antennae and legs (Figure 5A). Approximately 4.5 mm in length: HL: 0.5; EL: 0.54. Head: posterior margin of head in lateral view characteristically ‘sheared off’ with posterior side and ventral side at ca. 120°; eyes occupying approximately 1/4 of side of head; temples approximately 3.5× length of eyes; neck narrow. Antennae (Figure 5B): antennal insertions under “shelf” and therefore presumably not visible in dorsal view. All antennomeres elongate (Figure 5B); antennomere 1 expanded apically, equal in length to antennomeres 2 and 3 combined. Eyes occupying just under quarter of side of head; eyes longer than wide, anterior edge bordering posterior of shelf that conceals antennal insertions. Prothorax; pronotum sinuate in lateral view, superior marginal line inflexed under front angles and therefore not visible in dorsal view. Protibiae with large spur on inner margin, approximately halfway along length of tibia; two spurs present on apical area of protibia, one slightly longer than other. Protarsomeres 1 to 4 expanded with dense setation ventrally; protarsomere 5 was elongated, with several small setae only. Elytra unpunctured and glabrous, laterally with row of ca. 12 setae. Mesotibiae with 4(?) large apical spurs and several additional spurs positioned subapically. Abdomen cylindrical; intersegmental membrane with small brick-wall pattern; tergites III to VI (and possibly apical tergites) with distinct transverse microsculpture.

Etymology: The new species is named after Zi-Wei Yin (Shanghai, China), who facilitated our study of this material and in recognition of his contribution to Staphylinidae taxonomy and systematics.

## 4. Discussion

Here we have described three fossil species interpreted as the first known extinct representatives of the rove beetle subfamily Coomaniinae from the Upper Cretaceous Burmese amber, i.e., from the Late Cretaceous (earliest Cenomanian, ca. 99 Ma) [15]. The three fossil species described here share the following characteristics with extant *Coomania*: antennal bases concealed under “shelf”; neck very narrow; mesothorax with sub-basal ridge present; abdominal segments IV to VIII without paratergites; mesotibiae with multiple spurs. Unfortunately, several other characters, which could enforce the proposed systematic placement of the fossils, are not visible due to preservation. Other methods of morphological studies, such as micro-CT or synchrotron, may assist in making them visible in the future. There is a great difference in size between fossil *Coomania*, with the 9 mm long *C. megistos* being significantly bigger than the 4.5 mm long *C*. *yini* and the really small 3.5 mm long *C*. *enkarsios*. Other habitus features also vary to some extent. In extant *Coomania*, the species are uniform in size, all about 6 mm long.

The abundance and morphological diversity of Coomaniinae in Burmese amber suggest this lineage was common and diverse in the Late Cretaceous. This, along with the opposite pattern displayed by the rare and homogenous recent members of Coomaniinae, suggests this subfamily must have undergone notable extinctions, presumably at the border of the Cretaceous and Tertiary or later. It is notable that the sister group of Coomaniinae, Staphylininae (sensu [12]), clearly documented as fossils from the Early Cretaceous of China [14], has not yet been found preserved in Burmese amber. The only possible exception is undescribed specimens from Burmese amber, which are presumably a new genus of Diochini (Alfred F. Newton, personal communication). Recent Staphylininae are globally distributed but phylogenetically it comprises geographically heterogeneous lineages divided into the Southern hemisphere clade associated with Gondwana in origin and the Northern hemisphere clade stemming from Laurasia [17]. The absence of Staphylininae in Burmese amber may be due to their large size, which made them unlikely to fossilize in amber (all known Cretaceous Staphylininae fossils are large, and large Staphylininae are rare in Baltic amber too). Or in addition, they could be a rare lineage in the area of the Burmese amber deposition, or maybe absent entirely, for biogeographic reasons. The presence of extant undescribed species of *Coomania,* which we know from Australia (Queensland), Laos, Solomon Islands, Indonesia (Sumatra, Sumbawa), India (Nicobar Islands), Malaysia (Borneo Island, Penang Island), Philippines (Basilan, Leyte), Papua New Guinea and Thailand, may provide further evidence in support of the connection between the West Burma Block and Gondwana [18], which has been proposed as an explanation for some patterns shown between extant taxa and their relatives preserved in Burmese amber [19].

An alternative hypothesis, which is less likely given the available characters to observe in our fossils, but which is worth considering for further exploration, is that our fossils are in fact stem members of the Diochini lineage of Staphylininae that lost paratergites independently from Coomaniinae. In that respect, the abovementioned undescribed specimens from Burmese amber, which are presumably a new genus of Diochini (Alfred F. Newton, personal communication), are noteworthy and must be considered along with the abovementioned desired application of the micro-CT or synchrotron methods that would allow seeing more characters in our fossils.

## Data Availability

The data presented in this study are available in the present article.
